# A TRICk to Improve the Effectiveness of RIC: Role of Limb Temperature in Enhancing the Effectiveness of Remote Ischemic Conditioning

**DOI:** 10.3390/biology11010146

**Published:** 2022-01-17

**Authors:** Claudia Penna, Matteo Sorge, Francesca Tullio, Stefano Comità, Saveria Femminò, Mara Brancaccio, Pasquale Pagliaro

**Affiliations:** 1 Department of Clinical and Biological Sciences, University of Turin, 10043 Orbassano, Italy; claudia.penna@unito.it (C.P.); francesca.tullio@unito.it (F.T.); stefano.comita@unito.it (S.C.); 2 Department of Molecular Biotechnology and Health Sciences, University of Turin, 10126 Turin, Italy; matteo.sorge@unito.it; 3 Department of Medical Sciences, University of Turin, 10126 Turin, Italy; saveria.femmino@unito.it

**Keywords:** cardioprotection, remote ischemic conditioning, infarct size, reperfusion injury

## Abstract

**Simple Summary:**

Remote ischemic conditioning is a simple cardioprotective practice consisting in brief intermittent ischemia applied to a limb. Remote ischemic conditioning has been repeatedly validated in animal models. However, translation from animal experiments to clinics for remote ischemic conditioning has been disappointing. We have demonstrated that keeping the animal’s limb warm while performing intermittent ischemia reduces infarct size more effectively than cold intermittent ischemia; thus, we propose that a more accurate temperature control of the limb undergoing remote ischemic conditioning can increase the efficacy of this cardioprotective maneuver. A simple thermal blanket around the ischemic limb while performing remote ischemic conditioning could be an easy approach to test in humans, as it is simple and safe.

**Abstract:**

Background: Treatment of myocardial ischemia/reperfusion (IR) injury is still an unmet clinical need. A large variability of remote ischemic conditioning (RIC) protection has been reported; however, no studies have considered the temperature of the ischemic limb. We analyzed the effects of temperature on RIC protection. Methods: Left hind-limbs of anesthetized male mice were immersed in warm (40 °C, warm-RIC) or cold (20 °C, cold-RIC) water and subjected to a RIC protocol (4 × 5 min limb ischemia/reperfusion). In the control groups (warm-CTR or cold-CTR), the limbs underwent thermic conditions only. Isolated hearts underwent 30 min ischemia and 60 min reperfusion. A PI3K-inhibitor, LY294002 (5 µM), was infused in warm-RIC hearts before the IR protocol (warm-RIC LY). Infarct size was evaluated by nitro blue tetrazolium staining and expressed as the percent of risk area. Results: While cold-RIC did not reduce the infarct size compared to cold-CTR (51 ± 1.62% vs. 54 ± 1.07% of risk area, *p* = NS), warm-RIC (44 ± 1.13%) significantly reduced the infarct size with respect to either cold-RIC (*p* < 0.001) or warm-CTR (58 ± 1.41%, *p* < 0.0001). LY294002 infusion revealed the PI3K/Akt involvement in the warm-RIC protection. Infarct size reduction was abrogated by LY294002 pretreatment (warm-RIC: 44 ± 1.13% vs. warm-CTR 58 ± 1.41% *p* < 0.0001; vs. warm-RIC LY 54 ± 1.69% *p* = 0.0002). Conclusion: our study shows a remarkable difference between warm-RIC and cold-RIC in terms of infarct size reduction, supporting a pivotal role for limb temperature in RIC-induced cardioprotection.

## 1. Introduction

Despite the successes achieved against cardiovascular diseases, the prevalence of these diseases, including heart failure, continues to rise. In particular, ischemic disease is the leading cause of heart failure [1]; therefore, understanding the mechanisms of ischemia/reperfusion injury and finding new cardioprotective mechanisms is of paramount importance.

Currently, the only way to salvage ischemic myocardium from infarction and to limit infarct size is timely reperfusion. However, reperfusion per se adds an irreversible component of damage to the myocardium and thus contributes to infarct size (namely, ischemia/reperfusion injury, IRI) [2,3,4]. Despite improvements in the therapy of acute myocardial infarction over recent years, the incidence of myocardial infarction in the aged population is not declining and the number of survivors from acute myocardial infarction that go on to develop heart failure is increasing [5]. Therefore, treatment of IRI is still a major unmet clinical need and additional cardioprotective strategies are required [6,7].

Brief non-lethal episodes of myocardial ischemia and reperfusion protect the myocardium from the consequences of IRI by activating intrinsic molecular mechanisms [2,3,4]. Such protection by ischemic conditioning can be induced locally in the heart and in tissues remote from the protected organ—that is, so-called remote ischemic conditioning (RIC). RIC can be induced prior (remote preconditioning or pre-RIC), during (remote percoditioning or per-RIC) or after (remote postconditioning or post-RIC) a prolonged myocardial ischemia [2,3,4]. RIC induces the activation of the reperfusion injury salvage kinase (RISK) pathway (including PI3K/Akt pathway) and/or the survivor activating factor enhancement (SAFE) pathway within the heart, with some species differences in the involvement of both pathways [2,3,4].

Although evidence for the efficacy of RIC cardioprotective maneuvers has been described in all species tested so far, including humans [2,3,4], translation from animal experiments to clinical practice remains challenging and has been disappointing [8]. Additionally, large-scale randomized controlled trials have failed to confirm RIC-induced cardioprotection in patients [9]. A great variability in RIC protection among different laboratories has been described [10,11] and it is likely that the variability is greater than the actual perception of the phenomenon. Indeed, we should consider that RIC maneuvers lack standardization in terms of the number and duration of the brief cycles of ischemia and reperfusion, and that this may be one of the reasons for the variability in results.

Nevertheless, despite similarities in terms of number and duration, results can be still different in terms of efficacy of IRI limitation. Actually, in their interesting systematic review and meta-analysis, Bromage et al. reported a significant heterogeneity in effect size that could not be explained by any of the experimental variables analyzed by meta-regression [10]. From this analysis, it emerged that studies lack consistency in quality and study design. Indeed, other parameters can impact RIC maneuver efficacy. Intriguingly, to the best of our knowledge, no studies have considered the temperature of the ischemic limb among the variables to be controlled (limb temperature was not mentioned in the meta-analysis by Bromage et al. [10]). There is, therefore, a clear need for better-performed studies, with a particular emphasis on the detailed characterization of RIC protocols and investigation of the potential impact of limb temperature. 

To this aim, we used a standard pre-RIC protocol performed at two controlled limb temperatures, a cold temperature (20 °C; cold-RIC) and a warm temperature (40 °C; warm-RIC), to compare the cardioprotective efficacy of these two protocols in a model of cardiac ischemia/reperfusion. Here we demonstrate that it is enough to keep the ischemic conditioned limb warm (40 °C) to increase the cardioprotective efficacy of this practice, and that protection is causally due to PI3K/Akt activation within the myocardium.

## 2. Materials and Methods

### 2.1. Animals

Male FVB mice (n total 60) received humane care in compliance with Italian law (DL-116, 27 January 1992) and in accordance with the Guide for the Care and Use of Laboratory Animals published by the US National Institute of Health (NIH Publication No. 85-23, revised 1996). All efforts were made to minimize suffering. The local “Animal Use and Care Committee” approved the animal protocol (protocol no: E669C.44). The mouse is an animal species quite routinely used in the cardioprotection field [11], and in particular in RIC studies [10].

### 2.2. Remote Conditioning Protocol

Male FVB mice weighing between 25 and 35 g (10–15 weeks old) were given 500 U heparin and anesthetized with sodium pentothal (50 mg/kg) by intraperitoneal injections before being subjected to the Remote Ischemic Conditioning (RIC) protocol.

**Figure 1:**
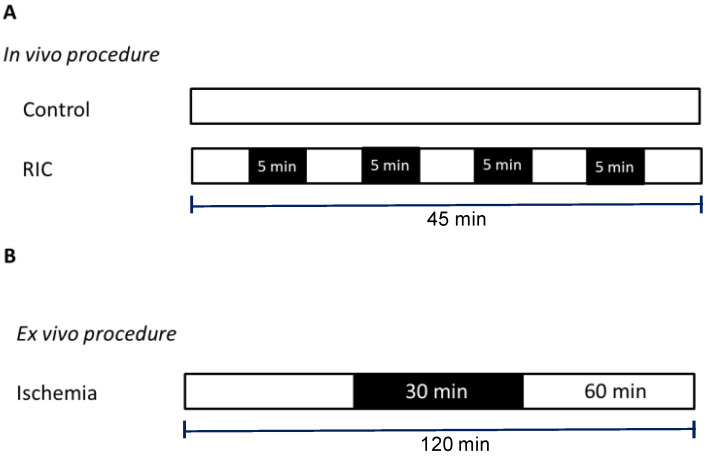
Experimental protocol. (**Panel A**): anesthetized mice underwent thermic procedures in which the left hindlimb was immersed in cold (20 °C) or warm water (40° C) only (Controls) or underwent a remote conditioning (RIC) protocol while the hindlimb was immersed in water. (**Panel B**): isolated mice hearts underwent a global normothermic (37 °C) ischemia/reperfusion procedure. Black boxes represent periods of ischemia; white boxes represent periods of normal perfusion.

Anesthetized animals had a small cuff placed around their left hind limb and were randomized to receive either the (A) Control protocol, consisting of a time period (45 min) with no cuff inflation or (B) RIC protocol, consisting of 4 cycles of 5 min cuff inflation to 200 mmHg with intermittent 5 min deflations [12]. The experiments in group A and B were performed with the studied limb immersed in water, whose temperature was kept at either 20 or 40 °C (Figure 1A).

Therefore, the experimental groups were:

1. Cold-Controls (cold-CTR): left hind limb was maintained at a temperature of 20 °C for 45 min without cuff inflation;
2. Warm-Controls (warm-CTR): left hind limb was maintained at a temperature of 40 °C for 45 min without cuff inflation;
3. Cold-RIC (cold-RIC): RIC was performed while the left hind limb was maintained at a temperature of 20 °C;
4. Warm-RIC (warm-RIC): RIC was performed while the left hind limb was maintained at a temperature of 40 °C.

At the end of the RIC protocol, the animals were sacrificed and the hearts were excised and perfused on a Langendorff apparatus, using the method described below.

### 2.3. Isolated Heart Perfusion Technique

The animals, while still anesthetized, were culled by cervical dislocation [13]. Hearts were rapidly excised and perfused retrogradely at 80 mmHg by the Langendorff technique with Krebs–Henseleit bicarbonate buffer containing (mM) NaCl 118, NaHCO_3_ 25, KCl 4.7, KH_2_PO_4_ 1.2, MgSO_4_ 1.2, CaCl_2_ 1.25 and Glucose 11. The buffer was gassed with 95% O_2_: 5% CO_2_. The temperature of the perfusion system was maintained at 37 °C. After 30 min of stabilization, 9 hearts were not used as they did not start to beat properly. 

The isolated hearts, after stabilization, were subjected to 30 min of global ischemia followed by 60 min of reperfusion (IR) at 37 °C (Figure 1B) [14]. A subgroup of warm-RIC hearts was also treated with 5 µM of the PI3K inhibitor LY294002 (warm-RIC LY) for 10 min before global ischemia. The dose of LY294002 was chosen on the basis of previous experiments [15].

The isolated heart allowed us to perform the IR protocol at well controlled temperatures to avoid influences on infarct size due to differences in body temperature [16]. Therefore, the groups differed only in ischemic limb temperature before undergoing heart isolation.

At the end of the perfusion period, hearts were removed from the perfusion apparatus and used for infarct size assessment [14].

### 2.4. Infarct Size Assessment

In brief, infarct areas were assessed with the nitro blue tetrazolium (NBT) technique in a blinded fashion, as previously described [14]. Immediately after reperfusion, hearts were removed from the perfusion apparatus and the ventricles were dissected by transverse sections into 2–3 slices. Following 20 min of incubation at 37 °C in 0.1% solution NBT (Sigma-Aldrich, St. Louis, MO, USA) in a phosphate buffer, unstained necrotic tissue was carefully separated from stained viable tissue by an independent observer, who was unaware of the protocols. The measurement of infarct size was performed first by planimetry, then immediately after by weighing the separated tissue by two different operators. Since a good correlation was found between the two measurements, we decided to report the data obtained by weighing, as they represented the mass of necrotic tissue more accurately. Since the ischemia was global and since we analyzed only the ventricles, the necrotic mass was expressed as a percentage of the analyzed ischemic tissue.

The apical slice of the ventricles, due to its small size, was not used for infarct size assessment, but was immediately frozen and subsequently analyzed by Western blot analysis [14].

### 2.5. Western Blotting 

Frozen samples were powdered in liquid nitrogen and lysed in Tris-buffered saline with 1% Triton X-100, plus Roche complete protease inhibitor cocktail, NaF 10 mM, PMSF 1 mM and Na3VO4 1 mM. Protein extracts were clarified by centrifugation for 20 min at 20,000× *g* at 4 °C [14].

Western blot band quantifications were performed with Image Lab software (Bio-Rad). Antibodies against Akt used: Akt (1:1000, #4691 Cell Signaling), P-Thr308 Akt (1:1000, #4056 Cell Signaling). As the secondary antibody: Anti-rabbit IgG-peroxidase antibody produced in goat (1:5000, #A6154, Sigma-Aldrich).

### 2.6. Statistical Analysis

All values are expressed as mean ± SE. Data were analyzed using one or two-way analysis of variance (ANOVA) followed by the Bonferroni post hoc test. For all analyses, a minimum value of *p* < 0.05 was considered significant. Statistical analyses were performed using GraphPad Prism 4 (GraphPad Software version 4.0).

## 3. Results

### 3.1. Warm-RIC Reduced the Extent of Myocardial Infarction More Effectively Than Cold-RIC

**Figure 2:**
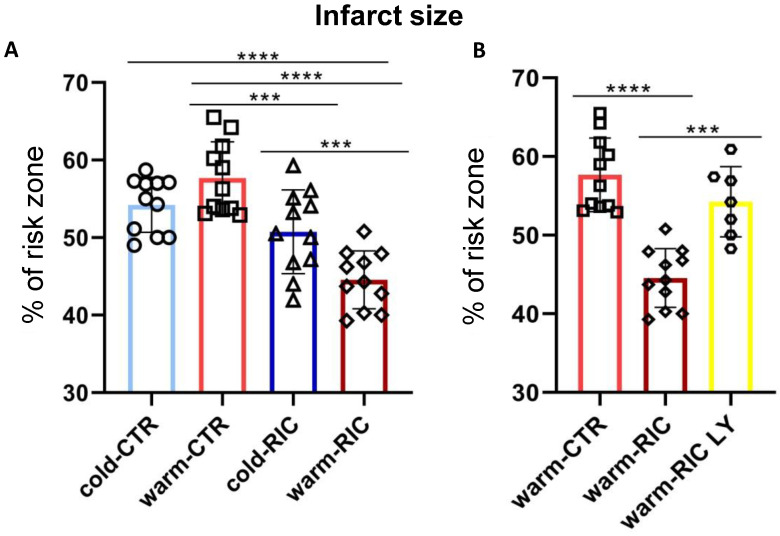
Infarct size (percentage of risk zone) of mice hearts. The isolated hearts of 5 different groups underwent ischemia/reperfusion protocols. The left hindlimb of anesthetized mice were subjected to remote ischemic conditioning (RIC) at cold (cold-RIC, n = 11) or warm (warm-RIC, n = 11) temperatures or exposed to cold (cold-CTR, n = 11) or warm (warm-CTR, n = 11) temperatures only. In a group of warm-RIC mice, LY294002 (5 µM; warm-RIC LY, n = 7) was infused into isolated hearts before the induction of ischemia. In (**A**) the first four groups are reported. In (**B**), warm-CTR and warm-RIC are reported again and placed next to the warm-RIC LY group to better highlight the statistical differences between these three groups (**** *p* < 0.0001; *** *p* < 0.001).

In both cold- or warm-CTR groups, the infarct size after 30 min ischemia and 60 min of reperfusion were 54 ± 1.07% and 58 ± 1.41% of risk zone (*p* = Not Significant (NS)), respectively. The cold-RIC did not significantly reduce the infarct size (51 ± 1.62% of risk zone, NS with respect to cold-CTR), while the warm-RIC significantly reduced the infarct size (44 ± 1.13%, *p* < 0.0001 with respect to warm-CTR; *p* < 0.001 with respect to cold-RIC; Figure 2A).

### 3.2. Warm-RIC Acted via the PI3K/Akt Pathway

To evaluate the potential involvement of the PI3K/Akt pathway as a protective pathway activated in response to RIC, additional warm-RIC hearts were subject to IR in the presence of the PI3K inhibitor LY294002 (5 µM). The administration of the inhibitor abolished the warm-RIC-induced protection. Indeed, after the LY294002 and IR protocol, the infarct size was 54 ± 1.69% of risk zone (*p* = 0.0002 compared to the warm-RIC; Figure 2B).

**Figure 3:**
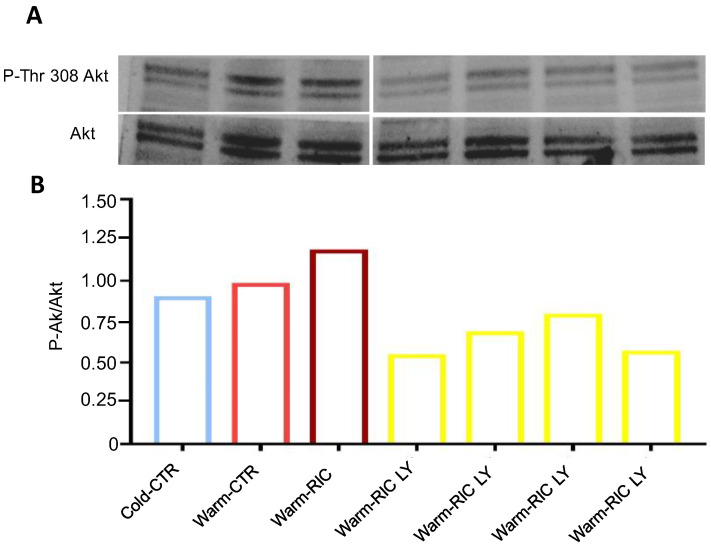
Western blot bands (**A**) and quantifications of phospho-Akt/Akt ratio (**B**) showing the effects of Akt phosphorylation inhibition following treatment with LY294002 in warm-RIC hearts compared to untreated warm-RIC and warm-CTR. Cold-CTR hearts are used as a reference group.

Western blot analysis was performed on hearts harvested from warm groups treated, or not treated, with LY294002 to investigate Akt phosphorylation. The analysis showed an increase in warm-CTR and warm-RIC in the phosphorylation of Akt and a clear reduction in the phosphorylation of Akt in the presence of LY294002 (Figure 3).

## 4. Discussion

Here we show that limb temperature influences the efficacy of RIC. Indeed, RIC performed at room temperature (20 °C), namely cold-RIC, induces a small non-consistent reduction of infarct size. However, RIC performed at warmer (40 °C) limb temperature, namely warm-RIC, induces more consistent and significant cardioprotective effects, as revealed by a significantly reduced infarct size. Results suggest that inhibition of PI3K activity reduces the cardioprotective properties of warm-RIC. This latter effect is in agreement with studies reporting a causal involvement of PI3K/Akt activation in the at-risk cardiac area via cardioprotective maneuvers in rodents [2,3,4]. We propose that warm-RIC is a stronger stimulus and may be considered a promising approach that deserves to be tested in a clinical scenario.

We started from the consideration that in the ischemic muscle, such as in RIC, metabolic changes occur that are very similar to those that occur in a muscle that is working, such as in physical exercise. In both cases, for example, the levels of ADP, AMP, Pi and reactive oxygen species (ROS) increase, and glycogen and pH levels decrease [17]. Both RIC and exercise can trigger beneficial mechanisms for the whole organism [18]. However, a substantial difference between muscle undergoing either ischemic preconditioning or exercise is that in the first case the muscle cools, while in the second case the muscle warms up. Therefore, we decided to compare RIC procedures performed at two different temperatures of the limb subjected to intermittent ischemia. We choose to compare a typical room temperature (20 °C) with the typical temperature that muscles reach during exercise (40 °C).

To our knowledge, nobody has ever taken the temperature of the limb into account in RIC studies. Since both RIC and exercise can trigger beneficial mechanisms for the whole organism, we hypothesized that heating the limbs while doing RIC maneuvers could enhance the protective efficacy of the conditioning protocol, making it more similar to physical exercise.

It is well known that the development of infarct size may be temperature dependent. In particular, lower core body or heart temperature may reduce infarct size after IR protocols. Some authors have also studied whether lowering core temperature may affect the cardioprotection attained by RIC [19]. In the study by Verdouw et al., RIC and infarct size experiments were performed at 36–37 or 30–31 °C. Pre-RIC was induced by a 15 min mesenteric artery occlusion or by a 15 min renal artery occlusion: both protocols were protective at lower core temperatures [19]. However, in this study, the myocardial IR protocol was conducted at a low temperature.

Temperature is among one of the most important variables to be controlled during ischemia/reperfusion studies, as it can greatly influence cell death and subsequent inflammatory processes. In vivo, in open chest acute experiments, the ischemic part will change temperature in an uncontrolled manner—depending mainly on room temperature, regardless of the use of a temperature-controlled operating table. Instead, in ex vivo Langendorff studies, the whole heart—including the ischemic part—is set to a well-controlled temperature. This avoids influences of temperature on infarct size [16]. Indeed, to avoid any influence of temperature directly on the myocardial IRI, we performed the pre-RIC in vivo and IR experiments after heart isolation, in an ex vivo model in which we can strictly control the heart temperature, which was set in all cases to 37 °C. Therefore, the infarct size reduction may not be attributed to a direct influence of temperature on IR injury, but to a potentiated mechanism starting from the ischemic limb, in which temperature plays a pivotal role.

The SAFE pathway, including the signal transducer and transcription activator (STAT)-3 and STAT5, and the RISK pathway, including PI3K/Akt, can play a central role in RIC cardioprotection—in both humans and mice [20]. Indeed, in STAT5 knockout mice it was found that RIC-induced infarct size reduction occurs via the PI3K/Akt pathway and the anti-apoptotic cascade, thus confirming the importance of RISK in rodents [21]. These aspects make mice an appropriate animal species for studying RIC. Here, we confirm the importance of the PI3K/Akt survival pathway in warm-RIC cardioprotection, using a specific PI3K inhibitor. Indeed, WB analysis confirms Akt phosphorylation by warm-RIC, which is clearly blunted by the PI3K inhibitor (Figure 3). 

Under our experimental conditions for all groups, only the leg was heated in vivo, and the entire heart was kept at identical temperatures during the ex vivo ischemia/reperfusion protocol. Therefore, it is unlikely that temperature played a direct role in the activation of the PI3K/Akt pathway. However, it is possible that one or more endogenous factors (more than 100 different signaling molecules may be released and involved in RIC [4,22]) are released from the warm-conditioned leg in greater quantities, reaching the threshold needed to activate the PI3K/Akt pathway in the myocardium. Future studies may ascertain this hypothesis.

RIC can be performed before ischemic events in programmed PTCA or cardio surgery intervention—that is, remote preconditioning—but it can be also used during an acute infarction (remote preconditioning) or after myocardial reperfusion (remote postconditioning) [2,3,4]. Here, we demonstrate the efficacy of warm-RIC for preconditioning. Nevertheless, we cannot rule out its superiority in preconditioning and postconditioning conditions. Additionally, these conditions may deserve to be tested in the future.

## 5. Conclusions

Warm-RIC obtained with transient ischemia of the hind-limb kept at 40 °C is more effective than cold-RIC (hind-limb kept at 20 °C) in reducing infarct size. Here, using a specific inhibitor for the PI3K/Akt pathway, we also confirmed that cardioprotective signal transduction causally involves an activation of Akt in the at-risk area of the mouse heart. 

Changing the whole-body temperature is an approach proposed to reduce IRI [23,24,25,26,27]. However, it is a challenging task. Yet, to change only limb temperature in humans can be easily achieved. For instance, a simple thermal blanket around the ischemic limb while performing RIC may be an easy approach for testing in humans. Therefore, our observation that warm-RIC is more effective than room temperature RIC (cold-RIC) is a promising result that deserves to be tested in a clinical scenario, as it is simple and safe.

## Data Availability

Data are available at request from pasquale.pagliaro@unito.it; claudia.penna@unito.it; mara.brancaccio@unito.it.
